# Editorial: Topological Neuroscience

**DOI:** 10.1162/netn_e_00096

**Published:** 2019-07-01

**Authors:** Paul Expert, Louis-David Lord, Morten L. Kringelbach, Giovanni Petri

**Affiliations:** Department of Mathematics, Imperial College London, London, UK; EPSRC Centre for Mathematics of Precision Healthcare, Imperial College London, London, UK; Department of Neuroimaging, Institute of Psychiatry, Psychology and Neuroscience, Kings College London, London, UK; Global Digital Health Unit, School of Public Health, Faculty of Medicine, Imperial College London, London, UK; Department of Psychiatry, University of Oxford, Oxford, UK; Department of Psychiatry, University of Oxford, Oxford, UK; Center for Music in the Brain, Aarhus University, Aarhus, Denmark; ISI Foundation, Turin, Italy; ISI Global Science Foundation, New York, New York, USA

**Keywords:** Topological data analysis, Neuroscience, Multiple scales, Higher order interactions

## Abstract

Topology, in its many forms, describes relations. It has thus long been a central concept in neuroscience, capturing structural and functional aspects of the organization of the nervous system and their links to cognition. Recent advances in computational topology have extended the breadth and depth of topological descriptions. This Focus Feature offers a unified overview of the emerging field of topological neuroscience and of its applications across the many scales of the nervous system from macro-, over meso-, to microscales.

From the early drawings of Ramon y Cajal to today, topological descriptions have played a central role in neuroscience. In recent years, thanks to advancements in both mathematical tools and data availability, the range and diversity of such descriptions are expanding rapidly, spanning theoretical, computational, and experimental approaches to brain connectivity. This Focus Feature on “Topological Neuroscience” aims at presenting the breadth of applicability of topological data analysis (TDA) methods in neuroscience across scales and modalities.

Computational topology offers new frameworks for both the analytical description and the understanding of brain function. A common denominator to these new tools is their ability to find meaningful simplifications of high-dimensional data. As such, TDA aims to capture mesoscale patterns of disconnectivity and explicitly encode higher order interactions, that is, interactions between more than two regions or components (Giusti, Ghrist, & Bassett, 2016). In addition to the description of the shape of spaces derived from neuroimaging data, topology might play an even more fundamental role in brain organization, as indicated by mounting evidence for how the brain encodes space and memories (Dabaghian, Mémoli, Frank, & Carlsson, 2012). Finally, the intrinsic robustness of TDA methods and the features they identify make them powerful candidates not only to characterize healthy brain function but also potentially as biomarkers for disease (Romano et al., 2014).

Recent seminal research has shown the potential and impact of topological approaches. Topological differences have been found at the population and individual levels in functional connectivity (Lee, Chung, Kang, Kim, & Lee, 2011; Lee, Kang, Chung, Kim, & Lee, 2012) in both healthy and pathological subjects. Higher dimensional topological features have been employed to detect differences in brain functional configurations in neuropsychiatric disorders and altered states of consciousness relative to controls (Chung et al., 2017; Petri et al., 2014), and to characterize intrinsic geometric structures in neural correlations (Giusti, Pastalkova, Curto, & Itskov, 2015; Rybakken, Baas, & Dunn, 2017). Structurally, persistent homology techniques have been used to detect nontrivial topological cavities in white-matter networks (Sizemore et al., 2018), discriminate healthy and pathological states in developmental (Lee et al., 2017) and neurodegenerative diseases (Lee, Chung, Kang, & Lee, 2014), and also to describe the brain arteries’ morphological properties across the lifespan (Bendich, Marron, Miller, Pieloch, & Skwerer, 2016). Finally, the properties of topologically simplified activity have identified backbones associated with behavioral performance in a series of cognitive tasks (Saggar et al., 2018).

This Focus Feature offers a unified overview of this emerging field of topological neuroscience and of its applications across many scales of the nervous system from macro-, over meso-, to microscales. First, Sizemore, Phillips-Cremins, Ghrist, and Bassett (2019) provide an accessible introduction to the language of topological data analysis and investigate its potential in structural and genetic connectivity datasets. Chung, Lee, DiChristofano, Ombao, and Solo (2019) focus instead on differences in whole-brain functional topology in a cohort of twins and propose a novel topological metric that captures the heritability of topological features. In the context of event-related fMRI, Ellis, Lesnick, Henselman-Petrusek, Keller, and Cohen (2019) investigate the feasibility of topological techniques for recovering signal representations under different conditions. At the mesoscopic scale, Babichev, Morozov, and Dabaghian (2019) propose a computational model to assess the effect of memory replays in parahippocampal networks on the development and stabilization of hippocampal topological maps of space. At an even smaller scale, Bardin, Spreemann, and Hess (2019) show that topological features of spike-train data can be used to understand how individual neurons give rise to network dynamics, and hence to classify topologically such emergent behaviors. From a methodological point of view, Patania, Selvaggi, Veronese, Dipasquale, Expert, and Petri (2019) build topological gene expression networks that robustly capture the relationships between genetic pathways and brain function. Finally, Geniesse, Sporns, Petri, and Saggar (2019) present open-source tools designed to explore graphical representations of high-dimensional neuroimaging data extracted using topological data analysis at the individual level and without spatial nor temporal averaging.

It is now high time to put topological neuroscience center stage and to bring together the growing but often separate communities involved in applied topological analysis. Still, numerous challenges and questions remain before TDA methods become widely accepted and can come to realize their full potential. Notably, more research is needed both in terms of contextualization and functional interpretation of topological features (Lord et al., 2016; Verovsek, Kurlin, & Lesnik, 2017), and of scalability and computability of some of these features (Otter, Porter, Tillmann, Grindrod, & Harrington, 2017). However, there are already encouraging signs coming from academic conferences and schools in related fields (e.g., Netsci, Conference on Complex Systems, Applied Machine Learning Days), where tracks or satellites dedicated to TDA methods are already being organized. In this context, and considering that network-based methods sit in the larger realm of TDA, the journal Network Neuroscience is a natural venue to nurture and grow topological neuroscience in the coming years.
